# Dietary practices and nutritional status of children served in a social program for surrogate mothers in Colombia

**DOI:** 10.1186/s40795-023-00685-1

**Published:** 2023-02-06

**Authors:** Patricia Acosta, Ricardo Rojas-Humpire, Edda E. Newball-Noriega, Wilter C. Morales-García, Jacksaint Saintila, Percy G. Ruiz Mamani, Salomón Huancahuire-Vega

**Affiliations:** 1 Aldeas Infantiles SOS Colombia, Programa Cartagena, Bolívar, Colombia; 2 Departamento de Ciencias Básicas, Escuela de Medicina Humana, Facultad de Ciencias de La Salud, Universidad Peruana Unión, Lima, Perú; 3 Grupo de Investigación P53, Escuela de Medicina Humana, Facultad de Ciencias de La Salud, Universidad Peruana Unión, Carretera Central Km 19, Ñaña, Lima 15, Peru; 4 Unidad de Salud Pública Escuela de Posgrado, Universidad Peruana Unión, Lima, Perú; 5 Escuela de Medicina Humana, Universidad Señor de Sipán, Chiclayo, Perú; 6 Facultad de Ciencias de La Salud, Universidad Privada San Juan Bautista, Lima, Perú

**Keywords:** Nutritional status, Dietary practices, Children, Social program, Malnutrition

## Abstract

**Background:**

Dietary practices are acquired in the family context and in turn can affect the health of family members, especially the nutritional status of children. The objective of this study was to determine the relationship between nutritional status and feeding practices in children from foster families served by the SOS Children's Villages program in Cartagena, Colombia.

**Methods:**

The study had a cross-sectional design. Through a non-probabilistic purposive sampling, 139 children from 0 to 5 years of age from the SOS Children's Villages Cartagena program were involved. The sociodemographic background of the participants was recorded and the nutritional status of the children was evaluated through anthropometric and biochemical measurements. Dietary practices were measured by means of a standardized questionnaire. Analyses were performed with Poisson regression models with robust variance. These regression models provided prevalence ratios (PR) with their respective 95% confidence intervals (95%CI).

**Results:**

Among dietary practices, it was observed that most families eat together at home (63.3%), watch television when they eat (55.4%), and have dietary norms (80.6%). Consumption of plant foods was predominantly high, especially vegetables (86.3%), fruits (92.1%), cereals (84.9%), root vegetables, and bananas (93.5%). Consumption < 4 times/week of soft drinks and industrialized juices increases 14.3 times the probability of low weight-for-height in the study population compared to the group that does not consume them. On the other hand, watching television while eating (PR: 2.82, 95%CI 1.32—4.69) and consumption of sweet snacks (PR: 2.24, 95%CI 1.03—4.87) increased the probability of low height-for-age; while having eaten norms at home decreased the probability of low height-for-age in the study population by 50%.

**Conclusion:**

It is necessary to develop and implement interventions such as preventive measures and early diagnosis of inappropriate feeding behaviors to ensure adequate nutritional status among children under 5 years of age.

## Introduction

Adequate nutrition is necessary for the development and growth of children; malnutrition reflects of poor economic and social development [1], including malnutrition as well as overweight and obesity, which constitute a serious public health problem in poor and developing countries, including among children [2] and is one of the main causes of morbidity and mortality in children under 5 years of age [3].

According to the World Health Organization (WHO), the main reasons for stunting in children are: inadequate complementary feeding and food insecurity, poor food quality, and low dietary diversity, as well as inadequate, infrequent and insufficient intake of essential nutrients for growth [4]. Globally, in 2016, approximately 155 million children under 5 years of age were underweight and 41 million were overweight or obese [5]. Latin America and the Caribbean are the most affected regions, with 45% of child deaths in these regions estimated to be due to malnutrition [3]. In Colombia, reports indicate that 2.5% and 12% of children under 6 years of age suffer from acute and chronic malnutrition, respectively [6], with the highest rates in the Caribbean region of the country [7].

Nutritional status is influenced by dietary practices, malnutrition is one of the important risk factors in the onset of many communicable and non-communicable diseases in both children and adults [1, 8]. Therefore, adequate nutrition during infancy ensures the growth, health and development of children to their full potential, while an imbalance between nutritional needs, intake, absorption and utilization of nutrients alters nutritional status [9].

Inadequate feeding practices are the main causes of child malnutrition [10]. These are characterized by excesses and/or deficits, for example: consumption of hypercaloric foods, with more saturated fats, trans fats, free sugars, salt, or sodium; in addition, the reduction of sufficient amounts of foods such as fruits, vegetables, and fiber, which lead to a unbalanced diet and are the main factor responsible for malnutrition in all its variations [11].

Eating practices are consolidated in the family, which has a strong influence on the acquisition of different eating behaviors and other factors that may affect the health of children [12]. Among the latter, sedentary behavior is identified as a risk factor for health, given that activities that imply adopting a physically inactive position in children, such as watching television, demand less energy expenditure and are associated with low metabolic rates, increased cardiovascular risk, overweight, obesity, diabetes, sleep disorders and inadequate eating habits [13, 14]. Malnutrition in early childhood is more common than is generally believed, especially in dysfunctional families, and it is common for children who grow up in an adequate family structure to have a better nutritional status than those who have a family pattern framed under any type of violence or in conditions of poverty [15]. In addition, low wages for adults and environmental factors related to food, psychosocial care, and infections can also affect child development [16].

WHO considers stunting among children under 5 years of age to be a public health problem when it is > 20% [17]. Among one of the governmental strategies in Latin America for the comprehensive care of children and adolescents in vulnerable situations are foster care programs for the alternative care of children and adolescents deprived of parental protection [18]. The Instituto Colombiano de Bienestar Familiar foster families program takes in children who have been separated from their parents, because they are in a situation of vulnerability, and one of its mottos is to ensure quality care for the child population [19]; however, despite the validation and training of direct care collaborators, the impact of foster families' feeding practices on the health of children in foster care is unknown.

For this reason, the objective of this research was to determine the relationship between nutritional status and dietary practices of children served in the foster care area of the SOS Children's Villages program in Cartagena, Colombia.

## Methodology

### Design, type of research and participants

This was a cross-sectional study. Through an intentional non-probabilistic sampling, 139 children aged 0 to 5 years from the SOS Children's Villages Colombia (Cartagena) program were included. This program is part of an initiative of the Colombian Institute of Family Welfare (Instituto Colombiano de Bienestar Familiar). The participants, besides belonging to this social support program, did not present any type of cognitive or motor disability and whose surrogate parents voluntarily accepted the participation of the minors in the study. All procedures were performed in accordance with the 1964 Helsinki Declaration and its later amendments. In addition, the study was authorized by the Program management and received the approval of the Research Ethics Committee of Peruvian Union University (Number 2021-CEUPeU-0042).

### Sociodemographic characteristics

A registration form was used to obtain information on the sociodemographic background of the participants: Sex, age, nationality of the mother and children, reasons for entering the program, vaccination schedule, entry documents, and enrollment in the General Social Security Health System. All these data were collected from the Program's registration system.

### Determination of nutritional status

The nutritional status of the children was determined through anthropometric and biochemical evaluations. The anthropometric measurements were taken at the Nutritional Clinic of the Children's Villages Program, Cartagena branch, by a professional dietician. Weight was measured using a calibrated SECA 834 electronic infant and toddler scale with a capacity of 20 kg (SECA®, Hamburg, Germany). Length was measured using a SECA 416 infant and toddler infantometer for stationary use with a measuring range of 33 to 100 cm (SECA®, Hamburg, Germany).

The weight/height and height/age indicators were determined according to the child growth patterns published by the World Health Organization (WHO) and adapted for the Colombian population by the Ministry of Health and Social Protection through resolution number 2465 of 2016 [20]. According to this resolution, the anthropometric classification and the cut-off points for the weight/height indicator are: Obesity (> + 3DE), overweight (> + 2 to ≤  + 3DE), risk of overweight (> + 1 to ≤  + 2DE), weight-for-height adequate (≥ -1 to ≤  + 1DE), risk of acute malnutrition (≤ -2 to < -1DE), moderate acute malnutrition (< -2 to ≥ -3DE) and severe acute malnutrition (< -3DE). For this work, the weight/height diagnosis was re-categorized into: low (risk of acute, moderate, and severe malnutrition), normal (adequate weight for height), and high (risk of overweight, overweight and obesity).

In relation to the height/age indicator, according to Colombian regulations, the anthropometric classification and cut-off points are: Adequate height for age (≥ -1DE), low height risk (≥ -2 to < -1DE), and low height for age (< -2DE). In this work, the height/age indicator was re-categorized into: Normal (adequate height for age) and stunting (risk of low height and low height for age).

The biochemical evaluation was through the measurement of hemoglobin levels, which was performed through HemoCue® (HemoCue AB, Hb 201 + , Angelhemo, Sweden).

### Determination of food practices

To evaluate the participants' eating practices, an adaptation of the questionnaire that evaluates eating practices, constructed and validated by Lera et al., 2013 in families of Chilean minors, was used. This instrument showed an internal consistency of 0.75, which is applied to the parents/guardians of the children [21]. The adaptation of this questionnaire was subjected to the assessment of 5 expert nutritionists specialized in child nutrition. In addition, a pilot test was conducted with 20 randomly selected parents and the reliability of the instrument was determined with a Cronbach's α coefficient of 0.8. The questionnaire applied had two sections: The first contains nine questions on the eating habits of the child and the family in general at home (do they eat together at home, do they watch television when they eat, are there feeding rules, what meals are eaten at home, who usually buys food at home, who prepares the child's food at home, is the child breastfed or breastfed, does the child like the food, are snacks with vegetables offered), with yes or no answer options. The second part is the section on the frequency of food consumption of children in the household. There are 20 questions on the frequency of consumption of milk and its derivatives, meats, foods of vegetable origin, and processed foods. The frequency of consumption was categorized into: no consumption (0 times per week), low consumption (1 to 4 times/week), and high consumption (5 times/week with 2 or more times/day).

### Data analysis

Data analysis was performed using the R programming language version 4.0.2. Depending on their categorical or numerical nature, the variables were described as absolute and relative frequencies or mean and standard deviation, respectively. These data were arranged in tables and graphs depending on the case. For the association analysis between feeding practices and nutritional status, Poisson regression models with robust variance were used. These regression models provided prevalence ratios (PR) with their respective 95% confidence intervals (95%CI). A *p* < 0.05 was considered statistically significant in the analyses.

## Results

The general characteristics of the study population are summarized in Table 1. In total, 139 children from the SOS Children's Villages, Colombia surrogate mother program were analyzed. Most of the participants were of Colombian nationality (76.3%) who were admitted for the reason of neglect due to negligence (47.5%), 48.2% were boys, and 51.8% were girls, with an average age of 30.5 ± 19 months. The nutritional evaluation showed that the diagnosis of weight/height and height/age was normal in most cases; however, a significant proportion of stunting (30.9%) and high weight-for-height (16.5%) were found. The average hemoglobin level of the participants remained within normal limits (> 11 g/dL).

**Table 1:** General characteristics of the population

Variables	Total (*n* = 139)
Age (months)	30.5 ± 19.0
Sex (%)	
Boys	67 (48.2)
Girls	72 (51.8)
Nationality (%)	
Colombian	106 (76.3)
Venezuelan	32 (23.0)
Egyptian	1 (0.7)
Reason for admission (%)	
Abandonment	18 (12.9)
Sexual Abuse	26 (18.7)
Neglect due to negligence	66 (47.5)
Child of a teenage mother in protection	5 (3.6)
Domestic abuse	24 (17.2)
Weight (kg)	12.7 ± 4.9
Height (cm)	86.7 ± 16.5
Diagnostic weight/height (%)	
Under	6 (4.3)
Normal	110 (79.1)
Elevated	23 (16.5)
Diagnosis height/age (%)	
Normal	96 (69.1)
Growth retardation	43 (30.9)
Hemoglobin (g/dL)	12.2 ± 0.5

Data were presented as absolute frequencies (%) or means ± SD

**Fig. 1:**
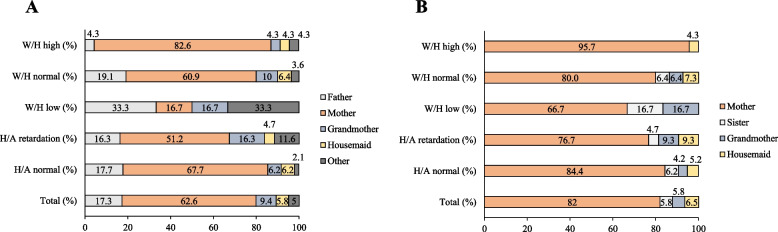
Changes in height/age and weight/height of children in the social program with respect to food procurement and preparation. **A** Foster family member who buys the food, **B** Foster family member who prepares the food. W/H, Weight-for-length; H/A, Height-for-age; Total (%), represents the household member who buys or prepares food

Dietary practices showed that most families eat together at home (63.3%), watch TV when they eat (55.4%), and have eating norms (80.6%); watching TV when they eat evidenced changes in frequency in stunting for age (Table 2) and underweight for height (Table 3) in the study population. On the other hand, norms for feeding presented a marked difference between the normal height-for-age and stunting groups. The consumption of breakfast, lunch, dinner, and snacks was predominant in all study groups. Both the purchase (62.6%) and the preparation of food (82%) was mainly done by the mother and tended to increase the weight-for-height of the children. However, in the low weight-for-height group, the father was the main provider of food (Fig. 1A and B). In all groups, it was evident that few snacks with vegetables were offered and they welcomed the food; on the other hand, more than 90% showed that they had not been breastfed.

**Table 2:** Dietary practices of the study population as assessed by the height/age relationship

**Food practices**		**Height/age**	
	**Total (*n* = 139)**	**Normal (*n* = 96)**	**Growth retardation (*n* = 43)**
Eating together at home (%)			
Yes	88 (63.3)	65 (67.7)	23 (53.5)
No	51 (36.7)	31 (32.3)	20 (46.5)
Watch TV when they eat (%)			
Yes	77 (55.4)	48 (50.0)	29 (67.4)
No	62 (44.6)	48 (50.0)	14 (32.6)
There are food standards (%)			
Yes	112 (80.6)	83 (86.5)	29 (67.4)
No	27 (19.4)	13 (13.5)	14 (32.6)
Meals provided (%)			
Breakfast, lunch, and snacks	5 (3.6)	2 (2.1)	3 (7.0)
Breakfast, lunch, dinner, and snacks	134 (96.4)	94 (97.9)	40 (93.0)
Breastfeeding (%)			
Yes	4 (2.9)	4 (4.2)	0 (0.0)
No	135 (97.1)	92 (95.8)	43 (100.0)
Is satisfied with the food (%)			
Yes	138 (99.3)	95 (99.0)	43 (100.0)
No	1 (0.7)	1 (1.0)	0 (0.0)
Offers snacks with vegetables (%)			
Yes	10 (7.2)	6 (6.2)	4 (9.3)
No	129 (92.8)	90 (93.8)	39 (90.7)

Data were presented as absolute frequencies (%)

**Table 3:** Dietary practices of the study population according to weight/height ratio assessment

	**Weight/height**		
**Food practices**	**Under (*n* = 6)**	**Normal (*n* = 110)**	**Elevated (*n* = 23)**
Eating together at home (%)			
Yes	5 (83.3)	68 (61.8)	15 (65.2)
No	1 (16.7)	42 (38.2)	8 (34.8)
Watch TV when they eat (%)			
Yes	4 (66.7)	62 (56.4)	11 47.8)
No	2 (33.3)	48 (43.6)	12 (52.2)
There are food standards (%)			
Yes	5 (83.3)	90 (81.8)	17 (73.9)
No	1 (16.7)	20 (18.2)	6 (26.1)
Meals provided (%)			
Breakfast, lunch, and snacks	1 (16.7)	4 (3.6)	0 (0.0)
Breakfast, lunch, dinner, and snacks	5 (83.3)	106 (96.4)	23 (100.0)
Breastfeeding (%)			
Yes	0 (0.0)	3 (2.7)	1 (4.3)
No	6 (100.0)	107 (97.3)	22 (95.7)
Is satisfied with the food (%)			
Yes	6 (100.0)	109 (99.1)	23 (100.0)
No	0 (0.0)	1 (0.9)	0 ( 0.0)
Offers snacks with vegetables (%)			
Yes	0 ( 0.0)	9 (8.2)	1 (4.3)
No	6 (100.0)	101 (91.8)	22 (95.7)

Data were presented as absolute frequencies (%)

**Fig. 2:**
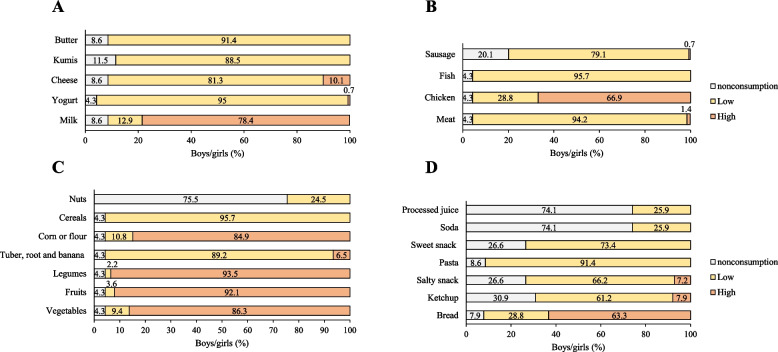
Frequency of food consumption among children in the social program. **A** Consumption of dairy products, **B** Consumption of foods of animal origin, **C** Consumption of foods of vegetable origin, **D** Consumption of processed foods

The frequency of food consumption evidenced a low consumption of dairy in general except for whole milk which had a 78.4% high consumption (Fig. 2A). Regarding the consumption of foods of animal origin, the majority presented low consumption, especially of sausages with 20.1% of non-consumption; however, chicken consumption was high at 66.9% (Fig. 2B). On the other hand, consumption of foods of plant origin was predominantly high, especially vegetables (86.3%), fruits (92.1%), cereals (84.9%), tubers, roots and bananas (93.5%) (Fig. 2C). In general, the consumption of processed foods was mainly low, except for of bread consumption, which was high at 63.3% (Fig. 2D).

Table 4 shows the relationship between nutritional status and dietary practices, showing that the consumption < 4 times/week of soft drinks and industrialized juices increased 14.3 times the probability of low weight for height in the study population compared to the group that did not consume them. On the other hand, watching television when eating (PR: 2.82, 95%CI 1.32—4.69) and consumption of sweet snacks (PR: 2.24, 95%CI 1.03—4.87) increased the probability of low height-for-age; while having eaten norms at home decreased the probability of low height-for-age in the study population by 50%.

**Table 4:** Relationship between nutritional status and dietary practices

Variables	RP	(IC95%)	*p*
**Weight/height (Underweight for height)**			
Soda Consumption (%)			
No consumption	1	reference	-
1–4 times/week	14.3	(1.72—40.3)	0.014*
Consumption of industrialized juices (%)			
No consumption	1	reference	-
1–4 times/week	14.3	(1.72—40.3)	0.014*
**Height/age (Low height for age)**			
Watching TV when eating (%)			
No	1	reference	-
Sometimes	2.82	(1.32—4.69)	0.005*
Yes	1.45	(0.80—2.61)	0.213
Feeding standards (%)			
No	1	reference	-
Yes	0.5	(0.27—0.97)	0.003*
Consumption of sweet snacks (%)			
No consumption	1	reference	-
1–4 times/week	2.24	(1.03—4.87)	0.042*

*PR* Prevalence radius

^*^p < 0.05, statistically significant by Poisson regression with robust variance

## Discussion

The nutritional status of children is conditioned by different factors: consumption of high-calorie dense foods, such as sugary drinks and snacks, exposure to television while eating, low purchasing power, and lack of nutritional knowledge of parents, the way food is prepared, among others [22]. The objective of the study was to determine the relationship between nutritional status and feeding practices in children cared for in the area of foster families by the SOS Children's Villages program in Cartagena, Colombia. The results show that having food standards conditions, a favorable nutritional status, while watching television while eating, consuming sweet snacks, industrialized juices, and soft drinks predispose to an unfavorable nutritional status for the development and growth of the children who were part of the study.

Stunting is a manifestation of suboptimal nutritional status and, in children, constitutes a major public health problem [23]. In our study, we found that a significant proportion of the children were stunted (low height-for-age). These results are similar to those reported in studies carried out in Colombia and other countries in the Latin American region [23, 24]. Moreover, findings from a similar study in children under 5 years of age found that a considerable proportion were stunted [25]. The causes of stunting are multifactorial. For example, the presence of infectious diseases can seriously contribute to the appearance of low height for age [26]. This may be due to increased energy expenditure and needs, which, in turn, may decrease appetite and increase nutrient loss, due to poor digestion, absorption, and disruption of metabolic and intestinal balance caused by vomiting associated with the infection [27]. In Colombia, access to public services, such as drinking water, is very limited [7]. The lack of drinking water is a problem that favors microbiological contaminants that lead to situations such as diarrhea and infectious diseases that can result in inadequate nutritional status, particularly underweight and stunted growth in children [28].

On the other hand, diet represents one of the main determinants of nutritional status in children. The influence of dietary intake on the growth of children is undeniable and well documented in the literature [26, 29, 30]. For example, healthy diets with a predominance of dairy products, vegetables, fruits, and whole grains are necessary to meet the nutritional requirements of children to ensure adequate growth and have a positive impact not only on weight but also on height [30]. However, an important aspect of chronic malnutrition or stunting in children is micronutrient deficiency during pregnancy, exclusive breastfeeding, and well-planned complementary feeding. Furthermore, there is usually a high consumption of an unbalanced and unbalanced diet, characterized mostly by high-calorie foods [31]. In the current study, it was found that the consumption of < 4 v/s of soft drinks, industrialized juices, and sweet snacks increased the probability of having low weight for height. These foods are mostly known as ultra-processed foods. Ultra processed foods and beverages are calorically dense, contain high amounts of unhealthy nutrients such as free sugars, sodium, and saturated fats, and are low in beneficial nutrients such as fiber and micronutrients [32]. These products may represent not only a risk factor for health (appearance of non-communicable diseases) but also for the nutritional status of children [33]. This could be due to the fact that, in these foods and beverages, there is not an adequate intake of macronutrients and micronutrients, such as proteins, healthy fatty acids, vitamins, minerals, and bioactive elements, essential for the growth and physical development of children [34]. Therefore, understanding which specific foods and nutrients are associated with children's growth is necessary to implement food-based intervention programs targeting parents, caregivers, and teachers to achieve better health and nutritional outcomes in children.

In general, children are attracted to television. In fact, the results of one study reported that more than 50% of children watch TV while eating [35]. Watching television while consuming food can have negative impacts on the nutritional status of children. Another relevant result of this study is that watching TV while eating represented a risk factor for low height/age in the study participants. Children who watch television are more likely to have an impaired anthropometric nutritional status [36, 37]. It is possible that this may be due to the mediating effect of children's food intake on the relationship between television viewing and anthropometric nutritional status [37]. Likewise, television viewing during family meals is associated with poorer outcomes in dietary intake and overall diet quality [36]. Inappropriate eating behaviors, characterized by the ingestion of certain food groups by children, generally occur when they watch television [38]. The more time spent watching television, the greater the intake of unhealthy foods rich in energy, saturated fats, free sugars, sodium, and less fruit and vegetables [39], which, in turn, has a clear impact on children's physical growth and development.

On the other hand, the existence of feeding norms at home favored a correct height for age in the present study; to date there is little information on this fact at the international level and even more so in the South American population. Thus, a study on parental eating behavior and eating styles related to nutritional status in schoolchildren showed that, in the regression models, control over eating, enjoyment of food, irritability, and responsiveness to satiety were statistically significant; among which control over eating through family guidelines had a strong relationship in the regression models [40].

### Limitations

It is important to emphasize that this study has some limitations that should be mentioned. The study did not measure dietary intake considering each food specifically and then associate it with nutritional status. It is suggested that future studies relate the link between the amount of food consumed or the size of the portions and the nutritional status of children. Furthermore, due to the cross-sectional design of the study, the results cannot suggest causal relationships between the variables evaluated. Therefore, the study of dietary practices and nutritional status of children in longitudinal and intervention research focused on improving dietary intake and avoiding anthropometric alterations may contribute to the understanding of the cause of the variables.

However, despite these limitations, we believe that the study is relevant because it provides solid findings on children under 5 years of age in Colombia, a population of great interest for public health. In addition, the results can be used as a reference for planning nutrition education programs and can also be used to encourage parents or caregivers to improve their eating habits and dietary intake and prevent anthropometric alterations of children in Colombian society and in other developing regions.

## Conclusion

This study concludes that most of the children had normal development as assessed by the anthropometric indicators weight-for-height and height-for-age, with a slight tendency to overweight and stunting. Watching television while eating, consuming sweet snacks, industrialized juices, and soft drinks predispose to an unfavorable nutritional state for development and growth, promoting low weight for height and stunting for age. On the other hand, having food standards conditions had a favorable nutritional status. In view of these results, improvements in the nutritional status of Colombian children and other similar developing regions require intensive efforts based on improving the feeding practices of all household members, particularly children under 5 years of age. This will be possible through the implementation of nutritional intervention programs aimed at parents and caregivers of children to promote practices that can ensure an adequate dietary pattern.

## Data Availability

The data and materials of the current study are available on reasonable request from the corresponding author.
